# Primaquine: the risks and the benefits

**DOI:** 10.1186/1475-2875-13-418

**Published:** 2014-11-03

**Authors:** Elizabeth A Ashley, Judith Recht, Nicholas J White

**Affiliations:** Mahidol-Oxford Tropical Medicine Research Unit (MORU), Faculty of Tropical Medicine, Mahidol University, Bangkok, Thailand; Centre for Tropical Medicine and Global Health, Nuffield Department of Medicine, University of Oxford, Oxford, UK

**Keywords:** Primaquine, G6PD deficiency, Malaria, Haemolysis

## Abstract

Primaquine is the only generally available anti-malarial that prevents relapse in vivax and ovale malaria, and the only potent gametocytocide in falciparum malaria. Primaquine becomes increasingly important as malaria-endemic countries move towards elimination, and although it is widely recommended, it is commonly not given to malaria patients because of haemolytic toxicity in subjects who are glucose-6-phosphate dehydrogenase (G6PD) deficient (gene frequency typically 3-30% in malaria endemic areas; >180 different genetic variants). In six decades of primaquine use in approximately 200 million people, 14 deaths have been reported. Confining the estimate to reports with known denominators gives an estimated mortality of one in 621,428 (upper 95% CI: one in 407,807). All but one death followed multiple dosing to prevent vivax malaria relapse. Review of dose-response relationships and clinical trials of primaquine in G6PD deficiency suggests that the currently recommended WHO single low dose (0.25 mg base/kg) to block falciparum malaria transmission confers a very low risk of haemolytic toxicity.

## Background

Primaquine is an 8-aminoquinoline, a descendant of the first generally available synthetic anti-malarial plasmoquine (plasmochin, pamaquine). The 8-aminoquinolines have unique anti-malarial properties, but they also pose particular safety problems. This has often divided medical opinion, and resulted in divergent treatment recommendations and prescribing practices. The 8-aminoquinolines kill mature gametocytes of *Plasmodium falciparum,* developing parasites of all species in the liver (causal prophylactic activity), the dormant hypnozoites of *Plasmodium vivax* and *Plasmodium ovale* (radical curative activity), and they have weak asexual stage activity (very weak for *P. falciparum*). The downside is that they cause haemolysis in people who are glucose-6-phosphate dehydrogenase (G6PD) deficient. This X-linked abnormality is very common in tropical areas with gene frequencies typically ranging from 3 to 30%. The renewed interest in malaria elimination, the rising concern that spread of artemisinin-resistant falciparum malaria could derail these elimination efforts and reverse recent substantial gains in reducing malaria morbidity and mortality, and the belated recognition that repeated relapse in vivax malaria is a major cause of morbidity (and in areas of high transmission, mortality), have focused renewed attention upon primaquine.

### Search strategy and selection criteria

References were extracted from an extensive literature search performed during a World Health Organization (WHO) evidence review of 8-aminoquinoline safety [1]. References were identified through searches of NLM PubMed for articles in English, French, Spanish, Italian, and German using the terms ‘primaquine’, ‘pamaquine’, ‘plasmoquine’, ‘G6PD’ and ‘G6PD deficiency’ and the limit ‘human’, with no time limit. Unpublished documents in the WHO archives and safety reports from the Uppsala Monitoring Centre were also reviewed.

### History

The first mass use of primaquine was in the Korean War. Over 250,000 US soldiers received 14-day radical curative regimens to eliminate long latency *P. vivax* infections [2]. Since the 1950s, primaquine and, in the Union of Soviet Socialist Republics (USSR), the closely related quinocide, have been used extensively to prevent seasonal long latency *P. vivax*, but the number of exposures is unclear. In the 1970s, some 28 million people in the Chinese Province of Jiangsu alone received mass preventive radical treatment regimens [3], which suggests that as many as 100 million people or more may have been treated in the entire country. In Azerbaijan, Tajikistan, Northern Afghanistan, and North Korea (DPR Korea) approximately eight million people received mass treatments with primaquine to prevent or eliminate *P. vivax* infections. These treatments were supervised and the population monitored for adverse events [4]. In Nicaragua, 1.9 million people received a three day regimen of chloroquine and primaquine to control and eliminate vivax and falciparum malaria [5]. Although radical five- to 14-day primaquine regimens have been recommended widely for over 50 years in tropical regions where *P. vivax* is prevalent, it is not known how many treatments have been administered. Since the resurgence of malaria in the early 1970s in India, which bears the majority of the global burden of vivax malaria, there have been approximately three million malaria cases reported annually, over half caused by *P. vivax* [6].

As a gametocytocide in *P. falciparum* infections, only one dose of primaquine is given (traditionally 0.5-0.75 mg base/kg, recently reduced to 0.25 mg base/kg) [7]. Again, although commonly recommended in Asia and the Americas, it is uncertain how many millions have received this treatment over the past 60 years. Primaquine has been little used in the private sector, and although it is a highly effective prophylactic and was used weekly by the US military in the Vietnam War [8], it has seldom been recommended to prevent malaria in travellers. Thus despite administration of primaquine to some 200 million people, arguments continue about whether, when and how to give it!

### Efficacy

The hypnozoitocidal activity of primaquine is predominantly a function of total dose administered; 3.5 mg base/kg (adult dose ~15 mg/day for 14 days) prevents >90% of long latency *P. vivax* relapses*,* whereas twice the dose (total 7 mg base/kg; adult dose 30 mg/day for 14 days) is required for short latency frequently relapsing infections in east Asia and Oceania. Poor adherence is an important cause of reduced effectiveness. There is no evidence for acquired resistance to the liver stage activity. Both the parasite burden (number of hypnozoites in the liver) and degree of acquired immunity are important determinants of the therapeutic response. Primaquine has potent gametocytocidal activity in *P. falciparum* infections and rapidly sterilizes the treated infection [9, 10]. Provided a drug with adequate asexual stage activity is given as well, this substantially reduces onward transmission. In a recent randomized, double-blind, placebo-controlled trial in eastern Uganda, 468 children aged one to ten years with uncomplicated falciparum malaria and normal G6PD enzyme function (as assessed by the fluorescent spot test) were randomized to receive artemether–lumefantrine, combined with either placebo or with a single dose of 0.1 mg/kg, 0.4 mg/kg, or 0.75 mg/kg primaquine base. The mean duration of gametocyte carriage was 6.6 days (95% CI 5.3-7.8) in the 0.75 mg/kg reference group, 6.3 days (5.1-7.5) in the 0.4 mg/kg primaquine group (p = 0.74), 8.0 days (6.6-9.4) in the 0.1 mg/kg primaquine group (p = 0.14), and 12.4 days (9.9-15.0) in the placebo group (p <0.0001). Thus 0.4 mg/kg was non-inferior to 0.75 mg/kg [11], and 0.1 mg/kg occupied an intermediate position in the dose-response relationship. The 8-aminoquinolines are substantially more active in reducing infectivity to mosquitos. Data from direct transmission blocking assessments from mosquito feeding studies predict that, given together with an effective artemisinin combination treatment (ACT), a single 0.25 mg base/kg dose gives maximal gametocytocidal effects, and this is now the recommended dose [7].

### Toxicity

**Figure 1:**
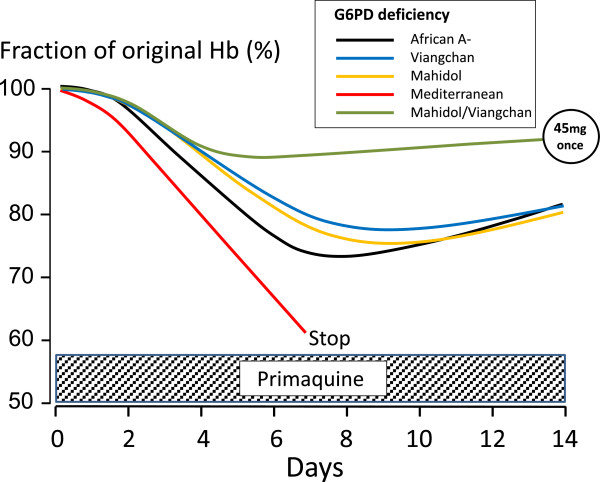
**Primaquine-induced haemolysis in adults with different G6PD variants during daily dosing.** Primaquine was given daily for 14 days at a dose of 30 mg/day in individuals with Mediterranean and African A-variants [18, 21] and 15 mg/day for Mahidol or Viangchan variants [22, 23]. The effects of a 45-mg single primaquine dose in individuals with either Mahidol or Viangchan variants are shown for comparison [24]. This figure uses data derived from different studies as referenced.

Dosing of primaquine is limited by abdominal discomfort at doses over 1 mg/kg. In general, primaquine is well tolerated at individual doses ≤0.5 mg base/kg if given together with food. Some methaemoglobinaemia is common, but very seldom is dangerous. The main adverse effect of primaquine is oxidant haemolysis. Although some red cell loss may occur in normal subjects, patients who are G6PD deficient are particularly vulnerable. It is the potential for toxicity in G6PD deficiency that has limited the use of primaquine. There are over 180 different genetic G6PD variants, nearly all conferring an unstable enzyme, which degrades more rapidly than the normal variant thereby rendering older red cells vulnerable to oxidant damage [12, 13]. The extent of haemolysis depends on the degree of G6PD deficiency and the dose and duration of exposure to primaquine. Two of the most prevalent G6PD variants represent ends of the severity spectrum with the Mediterranean variant (the main variant found in Europe, west and central Asia, and northern India) being amongst the most profound deficiencies, and the African A- variant (found in sub-Saharan Africa and in African-Americans) being amongst the mildest. Severe haemolytic reactions can still occur in G6PDA-, but they are much less frequent. Haemolytic risks vary widely as there is also substantial variability in G6PD activity between individuals with the same genotype, and even within the same individual over time. In less severe G6PD variants, primaquine-induced haemolysis typically becomes evident after one or two days’ exposure, when all the older erythrocytes’ oxidant defences (mainly reduced glutathione) have been depleted [14]. If primaquine is continued in subjects with the African A- variant then haemolysis lessens, and the haemoglobin starts to rise again despite further drug administration, as reticulocytes enter the circulation to replace the haemolysed cells (Figure 1) [15]. These young red cells contain five times more G6PD than the oldest red cells and so are relatively resistant to the haemolytic effect. However, further haemolysis does occur if higher doses of primaquine are given [16]. In contrast, in the severe Mediterranean variant, haemolysis continues if primaquine is not stopped and life-threatening anaemia may result (Figure 1) [17, 18]. Although the pathophysiology of primaquine haemolysis was dissected in detail in classic studies conducted in the 1950s and 1960s [14–20], several outstanding questions remain. Are patients with malaria at greater or lesser risk of haemolysis than healthy subjects? Does pre-existing hookworm or malaria-related anaemia reduce haemolysis? What exactly are the risks associated with the different therapeutic regimens? What reductions in haemoglobin are life threatening? There are also uncertainties over *P. vivax* relapse rates and latency intervals across much of the tropics which complicate therapeutic assessments, the methods for assessing gametocytocidal effects and their interpretation in clinical evaluations are disputed, and the community benefits from deployment of primaquine as a single dose gametocytocide have not been well characterized.

### Safety

#### Deaths

Only 14 deaths associated with primaquine have been reported over the past six decades of which 12 were from severe haemolysis (one was due to hepatic necrosis, and the cause of another was not stated) [1]. One fatality may have followed a single 45-mg dose [25]. All the other deaths followed multiple dose administrations. Haemolytic adverse effects, but no deaths, were reported from the mass treatments (MDA) in Jiangsu (>28 million treated [3]) and from the combined experience in Azerbaijan, Afghanistan, Tajikstan, and DPR Korea (>eight million treated [4]). G6PD screening was not performed during these MDAs but haemolysis was anticipated, and so education was provided and health services reinforced during the drug administrations. Excluding the Chinese data, the estimated risk for death associated with primaquine from all these reports is one in 621,428 with an upper 95% CI of one in 407,807. Including the Chinese data would make the risk four times lower. The reported deaths occurred mainly in countries with a minority of the global malaria burden, which does raise concern about the generalizability of this estimate.

### Severe adverse events

The most common severe adverse event (SAE) following primaquine is severe intravascular haemolysis with dark or black urine and mild jaundice. Allergic reactions and neuropsychiatric symptoms have been reported rarely in association with primaquine use. There are two serious consequences of severe haemolysis; life-threatening anaemia and haemoglobinuric renal failure. From 69 studies and additional case reports (excluding the MDA experiences) which evaluated primaquine adverse events, no SAEs were reported in G6PD-normal individuals, with the possible exception of one psychotic reaction. The 191 SAEs that were reported were in 25 individuals likely to have been G6PD deficient, and in 166 with proven G6PD deficiency (139 in case reports). The incidence of SAEs in the known G6PD-deficient group was 11.2% (27/241). Of all SAEs, 11.5% occurred after a probable overdose of primaquine, 75.9% with radical curative regimens for vivax malaria and 12.6% after administration of 30 or 45 mg primaquine in weekly prophylactic or radical curative regimens, or as a single-dose gametocytocide. Almost all the probable primaquine overdose (95.5%) SAEs were in children; most from a single case series of 21 Sri Lankan children aged two to 12 years hospitalized with acute intravascular haemolysis. All those tested for G6PD activity (17 children) were deficient [26].

In the 12 MDA programmes, 27 SAEs were reported, an estimated incidence of three SAEs per million. The majority of the SAEs were haemolysis, giving an estimated incidence of 1.8 episodes of severe haemolysis per million people receiving radical curative regimens of primaquine as MDA. In some MDA programmes given to almost 300,000 people in Azerbaijan (where the prevalence of G6PD deficiency, presumed Mediterranean variant, varied between 0 and 38.7%) and Kunduz Province, northern Afghanistan (Mediterranean variant prevalence 5-10%), an interrupted regimen was used, designed specifically so that haemolytic toxicity could be detected and managed. This was 15 mg primaquine (adult dose) once daily for four days, followed by three days without drug, then primaquine for a further ten days [27].

### Haemolysis following single-dose primaquine

Haemolysis is self-limiting as primaquine is eliminated rapidly (t_1/2_ ~ five hours). This is exploited in the once weekly 0.75-mg base/kg radical cure regimen recommended for patients with vivax malaria and ‘mild’ G6PD variants [2, 28], and the interrupted MDA regimens. Reticulocytosis following each dose compensates for haemolysis and the progressively younger red cell population becomes increasingly resistant to primaquine’s haemolytic effects. Few reports specifically examined the effect of single-dose primaquine on anaemia in the treatment of malaria, partly because malaria itself causes anaemia with a similar time course to that caused by oxidant haemolysis. Primaquine as a gametocytocide has usually been recommended in areas of low transmission where the haemoglobin nadir from malaria occurs around day 7. This coincides with the nadir following primaquine haemolysis. Given that the usual dose recommended (0.75 mg/kg) for the past 50 years was exactly the same as that recommended weekly for radical cure in G6PD-deficient patients [29], the risks have been assumed to be very low (the newly recommended 0.25-mg/kg gametocytocidal dose is three times lower [7]). G6PD testing has almost never been performed in this context. In 5,192 patients treated with mefloquine-sulphadoxine-pyrimethamine and primaquine (45 mg, average weight 50 kg: 0.9 mg base/kg) on the northwestern border of Thailand (G6PD-deficiency prevalence mainly Mahidol variant ~10%) no fatalities and no serious haemolysis was reported [30]. In a study in Tanzania, children received artesunate-sulphadoxine-pyrimethamine and 0.75 mg/kg primaquine, and in 15 children who were homo or hemizygous for G6PDA- deficiency there was a mean [95% CI] fall in haemoglobin on day 7 of -2.5 g/dL [-1.2 to -3.8 g/dL]. In one child, a G6PD A- heterozygote, the haemoglobin concentration fell from 8.3 to 4.8 g/dL [31]. In Myanmar (G6PD-deficiency prevalence 10-20%), in a randomized comparison of different ACTs in acute falciparum malaria, 397 patients received a single 0.75 mg base/kg dose of primaquine. Overall 30% of patients had pre-treatment haemoglobins <10 g/dL but no serious haemolysis (Hb ≤5 g/dL) was observed [32]. In Sardinia 19 healthy individuals with presumed Mediterranean type G6PD deficiency were given primaquine 45 mg and chloroquine 300 mg; the median [range] fall in haematocrit was 8% [6-19%] [18]. In Iran a single primaquine 0.33 mg/kg dose was given with chloroquine to six subjects with confirmed G6PD deficiency (likely Mediterranean variant but not genotyped) without malaria. All developed haemoglobinuria. The median [range] haemoglobin reduction was 2.3 [0.7-3.4] g/dL. None required a blood transfusion [17]. In a recent randomized trial of 468 Ugandan children with uncomplicated malaria who received artemether-lumefantrine followed by a single dose of primaquine 0.1, 0.4 or 0.75 mg/kg or placebo of whom 27% had some degree of G6PD deficiency, no children developed symptomatic anaemia [33].

### Dose-response relationship for haemolysis

**Figure 2:**
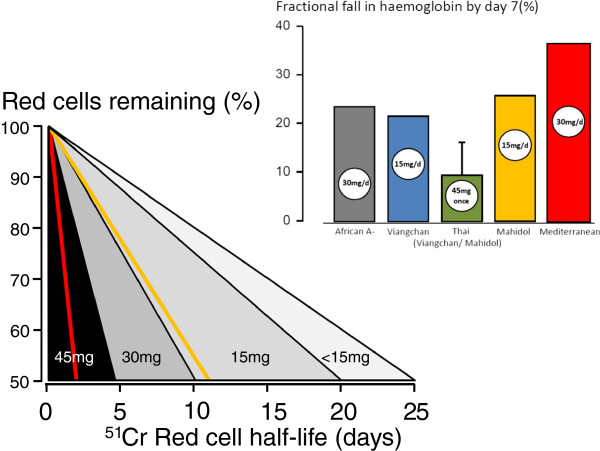
**G6PD-deficient red cell survival following daily primaquine dosing.** G6PD-deficient red cell survival was assessed by ^51^Cr labelling and transfusion into G6PD-normal healthy recipients (15 mg/day or 45 mg single dose in Mahidol or Viengchan variants, 30 mg/day Mediterranean variant, and a range of doses in African A- variant). The corresponding haematocrit reductions following continuous dosing (or in the green bar, a single dose) in G6PD-deficient subjects are shown in the inset [17, 21–24, 37].

Primaquine is metabolized *in vivo* via cytochrome P450 to reactive intermediates (mainly through CYP2D6) thought to mediate both anti-malarial and haemolytic effects [34]. These active intermediates have not been characterized definitively. Individuals with CYP2D6 genetic polymorphisms conferring reduced enzyme activity may have reduced primaquine efficacy [35, 36]. The relationships between primaquine dose, duration and haemolysis were characterized in detail for the G6PD A-variant in healthy adult volunteers. A clear dose-response relationship was evident; increasing daily doses up to 45 mg progressively shortened red cell survival (Figure 2) [21]. Dosing 45 mg daily could cause severe anaemia whilst 15 mg daily caused only mild anaemia. Taking 30 mg/day for 14 days in the African A- variant resulted in approximately the same degree of haemolysis as 15 mg/day in the Mahidol or Viengchan variants, with no further decline in haemoglobin during the second week of drug administration (Figure 1). These and other data are consistent with a ‘gene dosage’ effect as the African A- G6PD deficiency is less severe than the Mahidol and Viengchan variants, which in turn are less severe than the Mediterranean variant.

## Discussion and Conclusion

Primaquine is a very valuable anti-malarial drug, which is under-used. Concerns over potentially dangerous haemolytic toxicity along with the general unavailability of a simple test to identify patients at risk have substantially limited its deployment, and continue to do so. All patients with malaria haemolyse and all G6PD-deficient patients haemolyse additionally with primaquine treatment. The extent of haemolysis depends on the dose, duration and severity of deficiency. Whereas a single low dose is very likely to be safe, daily primaquine dosing for the radical cure of vivax malaria in a patient with severe deficiency risks potentially life-threatening haemolysis. Yet only 12 deaths from severe haemolysis have been documented over the past 60 years [1]. More may have gone unreported, although millions of people, many thousands of whom must have had severe variants of G6PD deficiency, received such regimens in MDAs without reported loss of life. The most likely explanation is simply that MDA recipients who suddenly felt ill and passed dark urine stopped taking primaquine.

For the physician contemplating radical curative treatment of *P. vivax* or *P. ovale* malaria, the correct approach is to test for G6PD deficiency. The NADPH ‘spot’ test identifies <30% of normal activity and should therefore identify all G6PD male hemizygotes and most female heterozygotes at high risk. Heterozygous females are genetic mosaics due to random X chromosome inactivation, also known as ‘Lyonisation’; as a consequence they may by chance have a predominance of deficient erythrocytes. Point-of-care tests with similar performance characteristics to the “spot test” have been developed recently [38]. For G6PD-deficient individuals the recommended options are to withhold primaquine, or in ‘mild deficiency’ to give 0.75 mg/kg once weekly for eight weeks. The once-weekly regimen was evaluated in a relatively small number of adults with the A- variant, and there is little published information on its use in other G6PD variants (most of which are more severe than A-). What should be recommended in the majority of *P. vivax*-endemic areas where G6PD-deficiency testing is not readily available? Multiple relapses cause substantial morbidity, interfering with development and educational performance in children, causing abortion and intra-uterine growth retardation in pregnancy and contributing to life-threatening anaemia in young children in higher transmission settings [39, 40] When relapse rates exceed 50% (e.g., in east Asia and Oceania), effective radical curative treatment more than halves the incidence of vivax malaria [41]. The benefits of radical cure are substantial for all patients, while the risks of iatrogenic haemolytic anaemia are borne by the minority who are G6PD deficient. If there is no available G6PD test the correct approach to patient management depends on the risks and the benefits i.e. the prevalence and severity of G6PD deficiency in the area and the dose regimen required for radical cure, the degree of anaemia and the availability of blood transfusion (i.e., the risk) as well as the probability and health impact of relapses (the benefit).

For the malaria control programme manager considering recommending primaquine as a gametocytocide in falciparum malaria, the assessment of risk is easier, whilst that of benefit is more difficult. The currently recommended 0.25 mg/kg dose is 14 to 28 times lower than the radical curative dose and two to three times lower than previously recommended gametocytocidal doses [7]. This low dose is associated with a red cell survival in the G6PD A- variant five times longer than with the previously recommended 0.75 mg/kg dose. Given the information on the safety of multiple dose radical cure, and the documented effects of higher doses in G6PD-deficient individuals, it seems highly unlikely that a single 0.25 mg/kg dose poses any significant haemolytic risk, even in individuals with severe G6PD deficiency. G6PD testing is, therefore, considered unnecessary. Assessments of the transmission blocking dose-response relationship based on indirect measures (gametocytaemia over time) provide an inconsistent and potentially misleading picture [42], probably because these measures underestimate and correlate poorly with reduction in infectivity [9, 10]. Direct dose-response assessments of infectivity to anopheline mosquito vectors suggest that primaquine 0.25 mg base/kg produces maximum gametocytocidal effects when given with an ACT [7]. The problem is that the treatment provides benefit to the community but no direct benefit to the treated patient. Whilst there is indirect evidence that blocking transmission of treated infections will reduce the incidence of malaria, proving it directly (in large, cluster-randomized, community-based trials) is very difficult. As transmission intensity increases the asymptomatic transmission reservoir increases, and the impact of reducing transmission in treated infections is progressively diluted. It is unclear at what level of transmission it is no longer “worth it” to give single dose primaquine. The corollary is that as transmission is reduced actively in an elimination programme every transmission source must be removed. As the community benefits depend on the proportion of patients who are treated, every effort should be taken to ensure that there is high coverage with single dose primaquine as a gametocytocide. One barrier to achieving this is the lack of availability of paediatric formulations.

The usual medical response to uncertainty is caution. However the struggle to contain and eliminate artemisinin-resistant falciparum malaria is a race against time, with potentially catastrophic consequences if we lose, so every approach to preventing the transmission of artemisinin-resistant parasites should be taken. It is essential to distinguish clearly the real risks of dangerous haemolytic anaemia associated with radical curative regimens of primaquine in vivax malaria from the considerably lower risk associated with giving a single small dose in falciparum malaria. This is why it is recommended that a gametocytocidal dose of primaquine should be added now to ACTs as part of regional elimination efforts [7].
